# Nitrogen Fertilization Alleviates Microplastic Effects on Soil Protist Communities and Rape (*Brassica napus* L.) Growth

**DOI:** 10.3390/microorganisms13030657

**Published:** 2025-03-14

**Authors:** Ge Wang, Maolu Wei, Qian Sun, Ting Shen, Miaomiao Xie, Dongyan Liu

**Affiliations:** 1 Key Laboratory of Land Resources Evaluation and Monitoring in Southwest, Sichuan Normal University, Ministry of Education, Chengdu 610101, China; 20221501032@stu.sicnu.edu.cn (G.W.); wml@stu.sicnu.edu.cn (M.W.); sq@stu.sicnu.edu.cn (Q.S.); st@stu.sicnu.edu.cn (T.S.); xmm@stu.sicnu.edu.cn (M.X.); 2 College of Life Sciences, Sichuan Normal University, Chengdu 610041, China

**Keywords:** rape, protist, microplastics, nitrogen addition, soil degradation

## Abstract

Agricultural plastic mulch enhances crop yields but leads to persistent microplastic contamination in soils. Concurrently, nitrogen (N) fertilization and atmospheric deposition profoundly reshape microbial ecosystems. This study examined the individual and interactive effects of polyethylene microplastics (PE, 1% *w*/*w*) and nitrogen addition (N, 180 kg ha^−1^ yr^−1^) on soil protist communities and rape (*Brassica napus* L.) productivity. High-throughput sequencing and soil–plant trait analyses revealed that PE alone reduced the soil water retention and the rape biomass while elevating the soil total carbon content, C/N ratios, and NH₄⁺-N/NO₃^−^-N levels. Conversely, N addition significantly boosted the rape biomass and the chlorophyll content, likely through enhanced nutrient availability. Strikingly, the combined PE_N treatment exhibited antagonistic interactions; protist diversity and functional group composition stabilized to resemble the control conditions, and the rape biomass under the PE_N treatment showed no difference from the CK (with basal fertilizer only), despite significant reductions under the PE treatment alone. Soil nutrient dynamics (e.g., the SWC and the C/N ratio) and the protist community structure collectively explained 96% of the biomass variation. These findings highlight the potential of nitrogen fertilization to mitigate microplastic-induced soil degradation, offering a pragmatic strategy to stabilize crop productivity in contaminated agricultural systems. This study underscores the importance of balancing nutrient management with pollution control to sustain soil health under global microplastic and nitrogen deposition pressures.

## 1. Introduction

Agricultural plastic mulch is an indispensable tool in modern agricultural systems that significantly enhances crop yields and improves water use efficiency [1,2,3]. However, improper recovery and the degradation of these plastic films leads to the accumulation of microplastic residues in soils, posing a growing environmental concern [4,5]. Once present, these microplastic fragments alter key soil physicochemical properties, such as reducing the water-holding capacity and porosity while disrupting critical ecological processes, like nitrogen (N) cycling [6]. Microplastics affect soil nitrogen cycling by inhibiting microbial activity and altering nitrogen transformation processes [7,8]. However, the interaction between microplastics and soil nitrogen remains unclear.

Simultaneously, agricultural ecosystems experience elevated nitrogen deposition owing to intensified fertilization practices and atmospheric inputs from fossil fuel combustion [9]. This excess nitrogen impacts soil microbial dynamics by introducing anthropogenic pressures that alter microbial composition and functional diversity [10]. While microbial-mediated nitrogen cycling has been studied extensively [11,12,13,14], the interactive effects of microplastic pollution and nitrogen deposition on soil eukaryotic communities, especially protists, remain largely unexplored. Protists enhance soil health by regulating bacteria and promoting nutrient cycling and plant growth [15,16]. Moreover, protists directly contribute to nitrogen transformation, providing essential functions in agricultural ecosystems [17,18,19]. Understanding how microplastics and nitrogen fertilizers jointly influence protist communities is essential to disentangle their broader impacts on soil health and crop productivity.

Most studies have focused on how microplastics affect bacterial and fungal communities in soil processes [20,21,22]. For instance, microplastics have been shown to inhibit enzymatic activities in bacteria and shift fungal community structures toward stress-tolerant taxa [21]. As key components of the soil microbiome, protists offer remarkable species and functional diversity, making them essential indicators of soil quality [23]. Typically acting as predators in the food web, protists release nutrients through microbial grazing, which plants then absorb to promote growth [24]. They also help regulate soil health by consuming harmful bacteria, fungi, and viruses, thus mitigating pathogen damage, reducing secondary metabolite production, and modulating bacterial functions [25]. Moreover, protists shape the rhizosphere microbial community and facilitate the transfer of organic nitrogen to plants via arbuscular mycorrhizal (AM) hyphae [26], playing a vital role in nutrient cycling and microbial community assembly [27]. Recent evidence further highlights that microplastics disrupt soil ecosystems by impairing plant–microbe–soil interactions, affecting roots, symbiosis, and metabolites [28,29,30]. However, the interactions between microplastics, nitrogen deposition, and protist diversity remain poorly characterized [30,31,32]. Protists mediate nitrogen mineralization, carbon turnover, and pathogen suppression, yet their responses to microplastic pollution and nitrogen enrichment are unknown [33,34]. This gap is critical in intensive agricultural systems, such as those with high rape production, where soil degradation and nutrient imbalances threaten long-term sustainability.

This study aimed to elucidate the combined effects of polyethylene (PE) microplastics and nitrogen addition on soil protist communities and rape performance in a nitrogen-enriched agricultural system. Using the high-throughput sequencing of 18S rDNA and soil–plant trait analyses, we hypothesized that (1) microplastic and nitrogen addition independently and interactively alter soil physicochemical properties, and (2) these changes significantly restructure protist communities, ultimately influencing crop productivity. By addressing the combined impacts of microplastics and nitrogen deposition, this study provides new insights into mitigating soil degradation and developing sustainable agricultural management strategies.

## 2. Materials and Methods

### 2.1. Soil and Microplastic Material Preparation

Soil samples were collected on 25 February 2023 from Qiusi Farm (30.34° N, 104.11° E), located at the Chenglong Campus of Sichuan Normal University, China. The site is situated in a subtropical humid monsoon climate zone, with annual temperatures of 14–22 °C, a mean annual precipitation of 771.8 mm, and cumulative annual temperatures of 4600–5000 °C. Soil collection was focused on the plow layer (0–20 cm depth) using a rotary tiller. Triplicate replicates, totaling approximately 100 kg of soil, were randomly extracted, pooled to form a composite sample, and transferred into sterile, air-tight polyethylene bags. The soil was air-dried at room temperature, sieved through a 2 mm stainless-steel mesh, and analyzed for the pH, water-holding capacity, and C/N ratio, with the remaining soil stored for pot experiments. The microplastic material consisted of polyethylene (PE) powder from the JieCheng Plastic and Chemical Company Limited (Dongguan, Guangdong, China). The PE particles had densities of 0.94–0.96 g/cm^3^ and diameters of 25–150 μm. The PE powder was stored in sealed sterile containers under dry conditions until use.

### 2.2. Experimental Design

The pot experiment, initiated on March 1, 2023, included four treatments to evaluate the effects of microplastic addition and nitrogen fertilization on rape growth: (1) control (CK): with basal fertilizer only; (2) microplastic addition (PE): polyethylene (PE) powder pre-mixed at 1% (*w*/*w*) of the total soil weight [35,36]; (3) nitrogen addition (N): additional nitrogen fertilizer, equivalent to 108 mg [37,38]; and (4) combined microplastic and nitrogen (PE_N): including both PE powder (1% *w*/*w*) and nitrogen fertilizer at 108 mg in each pot. All the treatments received basal fertilizer consisting of nitrogen (N), phosphorus (P), and potassium (K), applied at 60 kg N·hm^−2^, 30 kg P_2_O_5_ ·hm^−2^, and 30 kg K_2_O·hm^−2^, respectively [39,40]. Based on the treatment conditions, we homogenized the microplastics and base nitrogen fertilizers, either separately or together, prior to the experiment. A total of 64 pots were set up, with each pot (12 cm top diameter, 13 cm height, and 9.5 cm bottom diameter) containing 1 kg of dry soil, providing 16 replicates per treatment group. The soil for each treatment was thoroughly mixed and distributed into standardized pots. Two to three rapeseed seedlings (approximately 2 cm in height) were transplanted to the pots, and additional urea, as a nitrogen source, was applied in three equal splits over the 60-day growth period. By simulating the average precipitation during the rapeseed growing season and calculating the appropriate water volume, the seedlings in the pots were irrigated every 3–5 days in an open glasshouse. Key plant and soil indicators were measured at the end of the experiment.

### 2.3. Sample Collection and Soil Physicochemical Property Determination Methods

Destructive sampling was conducted on 5 June 2023, 60 days after rape transplantation. A total of 64 plant and 16 soil samples were collected. The plant samples were deactivated at 105 °C for 30 min, then dried at 80 °C for 48 h to determine the moisture content. Meanwhile, the soil samples were directly dried at 105 °C for 24 h. Plant measurements included the height, root diameter, leaf area, chlorophyll (a, b) content, root vigor (TTC method), and fresh weights of the stems, leaves, and roots. The soil samples were divided for microbial community analyses and physicochemical assessments. The soil properties measured included the pH (25 g soil: water, 1:2), total carbon (TC, potassium dichromate oxidation), total nitrogen (TN, Kjeldahl digestion), ammonium (NH₄⁺-N), nitrate nitrogen (NO₃^−^-N, 2 mol/L KCl extraction, indophenol blue method), and available phosphorus (AP, sodium bicarbonate extraction, molybdenum–antimony method).

### 2.4. High-Throughput Sequencing and Bioinformatics Analysis

Soil genomic DNA was extracted using a laboratory-made soil DNA extraction kit [41]. The DNA integrity was verified by 0.7% agarose gel electrophoresis, while the DNA concentration and purity were assessed using the OD260/OD280 and OD260/OD230 ratios on a NanoDrop 2000 spectrophotometer (Thermo Fisher Scientific, Wilmington, NC, USA). The V9 region of the 18S rRNA gene was amplified using primers Euk1391f (5′-GTACACCGCCCGTC-3′) and EukBr (5′-TGATCCTTCTGCAGGTTCACCTAC-3′) [42]. The PCR products were sequenced on Illumina MiSeq PE250 platform (Majorbio Bio-Pharm Technology Co., Ltd., Shanghai, China), with raw sequences processed through the DADA2 pipeline in QIIME2 [43]. Following quality control, the number of sequences retained per sample ranged from 56,478 to 156,928. The sequences were rarefied to a minimum sequence depth before further analysis with the vegan package in R (4.3.3). Sequence alignment was performed using the Protist Ribosomal Reference database (version 4.14). The obtained sequences were classified into three main functional types based on previous studies: consumers, parasites, and phototrophs [44,45].

### 2.5. Statistical Analysis

Both the plant and soil samples were set up with four biological replicates. The statistical comparisons of each physicochemical parameter were performed using a non-parametric Kruskal–Wallis test (with a significance level of *p* < 0.05) combined with Dunn’s multiple comparisons post-hoc test in GraphPad Prism (10.1.2). The protist α-diversity was compared among groups (CK, N, PE, PE_N) using a Wilcoxon test and visualized in GraphPad Prism (10.1.2). Soil physicochemical properties were analyzed using a one-way ANOVA with Duncan’s test (*p* < 0.05). The community composition was analyzed using NMDS (Bray–Curtis dissimilarity) and PERMANOVA to assess nitrogen, PE microplastics, and interaction effects. These analyses used the “vegan” package in R (4.3.3). The relative contributions of the protist community and soil properties to the rape biomass were analyzed using a generalized linear mixed model using the glmm.hp package in R (4.3.3).

## 3. Results

### 3.1. Rape Biomass and Physiological Characteristics

**Figure 1:**
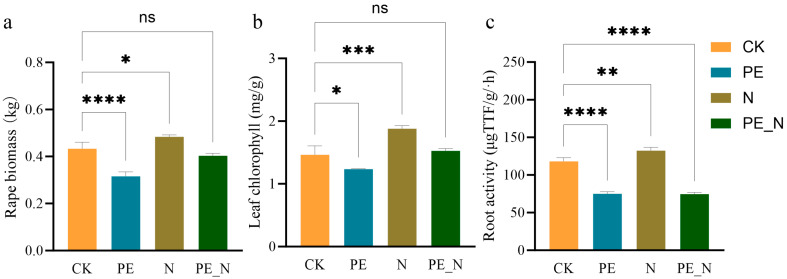
Rape biomass (**a**), leaf chlorophyll (**b**), and root activity (**c**) in different treatments. Values represent mean ± SE (n = 4). The ns indicates no significant difference and an asterisk (*) indicates a significant difference, according to the Kruskal–Wallis test with the Dunn post-hoc test, with the significance levels defined as follows: * *p* < 0.05, ** *p* < 0.01, *** *p* < 0.001, and **** *p* < 0.0001.

The biomass and physiological characteristics of rape exhibited significant variation across the different treatments, as illustrated in Figure 1. The biomass was markedly reduced under the PE treatment compared to the control (CK) (*p* < 0.0001). In contrast, the N treatment produced the highest biomass among all the treatments, showing a significant increase relative to both the CK and the PE treatments. However, the combined PE_N treatment resulted in a significant reduction in biomass compared to the N treatment alone (*p* < 0.05). The leaf chlorophyll content also varied significantly among the treatments. Specifically, the N treatment led to a significant increase in the chlorophyll content compared to the CK (*p* < 0.01), while the PE treatment caused a significant decrease relative to the CK (*p* < 0.01). Notably, no significant difference in the chlorophyll content was observed between the CK and the PE_N treatment. Root activity was profoundly influenced by the treatments. The PE treatment significantly reduced root activity compared to the CK (*p* < 0.0001). Similarly, the PE_N treatment exhibited significantly lower root activity than the CK (*p* < 0.0001) and N (*p* < 0.01) treatments. Conversely, the N treatment maintained root activity levels comparable to those of the CK, indicating that nitrogen addition alone did not adversely affect the root physiological performance.

### 3.2. Changes in Soil Physicochemical Properties

Changes in soil physicochemical properties under the CK, N, PE, and PE_N treatments were analyzed (Table 1). The PE_N treatment significantly reduced the SWC by 30% compared to the CK (*p* < 0.05), while the PE treatment caused a non-significant reduction of 13%. In contrast, N addition slightly increased the SWC by 4.3% relative to the CK, although this effect was not statistically significant. The soil pH exhibited minimal variation across the treatments. The PE treatment resulted in the highest pH level, while both the PE_N and N treatments slightly reduced the pH compared to the CK. However, none of these differences in the soil pH were statistically significant. The TC content was significantly higher under the PE (47% increase) and PE_N (77% increase) treatments than under the CK (*p* < 0.05). Although N addition also increased the TC content, this change was not statistically significant. The TN content exhibited no significant differences across the treatments. The TN content showed no significant differences across the treatments. While TN levels were marginally higher under the N treatment than under the CK, both the PE and PE_N treatments yielded slight reductions, although the changes were not statistically significant. The C/N ratio was significantly increased under the PE (49%) and PE_N (79%) treatments compared with the CK (*p* < 0.05). The N treatment also resulted in an elevated C/N ratio, but this change was not statistically significant. The AP level decreased across all the treatments compared with the CK. The PE_N treatment caused the greatest reduction (−24%), followed by the PE (−17%) and N (−14%) treatments. However, the reduction in AP under the PE and N treatments was not statistically significant. The NH_4_^+^-N content was significantly elevated under the PE_N treatment, with a 16% increase compared to the CK (*p* < 0.05). The NH_4_^+^-N levels also showed slight increases under the PE and N treatments, although these changes were not statistically significant. The NO_3_^−^-N content was significantly higher under the N and PE_N treatments than under the CK (*p* < 0.05), with the PE_N treatment producing the largest increase (16%). Conversely, the PE treatment caused a slight decrease in the NO_3_^−^-N content, but this change was not statistically significant.

### 3.3. Changes in the Soil Protist Diversity and the Community Composition

**Figure 2:**
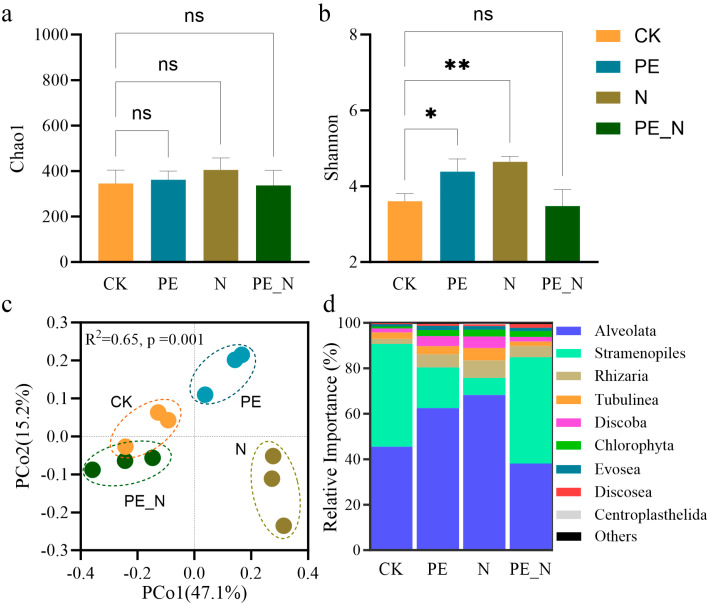
Soil protist Chao1 (**a**) and Shannon (**b**) indices, PCoA community analysis at ASV level based on Bray-Curtis dissimilarity (**c**), and relative abundance of dominant divisions (**d**) under different treatments. The ns indicates no significant difference and anasterisk (*) indicates a significant difference, according to the Kruskal-Wallis test with the Dunn post-hoc test, with the significance levels defined as follows: * *p* < 0.05 and ** *p* < 0.01.

The Chao1 and Shannon diversity indices revealed distinct patterns among the treatments (Figure 2a,b). For the Chao1 index, no significant differences were observed across the treatments (CK, PE, N, and PE_N), although the N and PE treatments showed slightly higher values than the CK. In contrast, the Shannon index demonstrated significant changes in protist diversity. Both the PE and N treatments significantly increased protist diversity compared to the CK (*p* < 0.05 and *p* < 0.01, respectively), whereas protist diversity was significantly decreased under the PE_N treatment compared to the N treatment (*p* < 0.01). A principal coordinate analysis (PCoA) of the protist community composition indicated significant differences among the treatments (Figure 2c; Adonis, *p* = 0.001), with the first two axes explaining 62.3% of the total variation (PCo1 = 47.1%, PCo2 = 15.2%). The samples were clustered into four distinct groups corresponding to each treatment, highlighting the unique effects of the treatments on the protist community structure. A relative abundance analysis revealed marked differences in the composition of dominant protist divisions across the treatments (Figure 2d). In the CK treatment, *Alveolata* and *Stramenopiles* were the dominant groups. The PE treatment increased the relative abundance of *Alveolata* compared to the CK, whereas the N treatment reduced *Alveolata* and increased *Stramenopiles*. The PE_N treatment resulted in a distinct community composition, with significantly altered proportions of the dominant divisions compared to the other treatments. Additionally, some protist divisions, such as *Rhizaria* and *Tubulinea*, exhibited varying patterns of relative abundance across the treatments.

### 3.4. Changes in the Functional Group of the Soil Protist Community

**Figure 3:**
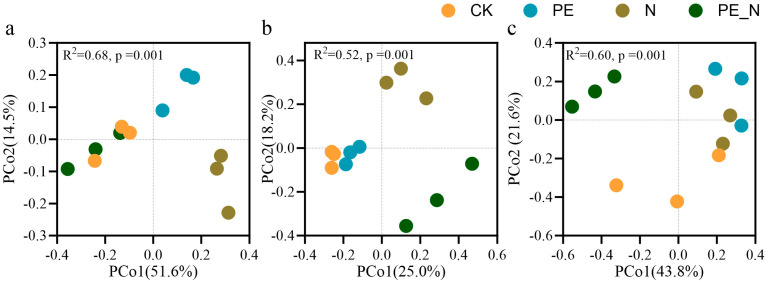
PCoA community analysis at ASV level, based on Bray-Curtis dissimilarity of consumers (**a**), phototrophs (**b**), and parasites (**c**) under different treatments.

The PCoA analysis revealed significant differences in the community composition of various functional groups under the treatments (Figure 3). For the consumer community (Figure 3a), the PCoA results indicated a clear separation among the treatments (R^2^ = 0.68, *p* < 0.001), with the first two axes explaining 66.1% of the total variation (PCo1 = 51.6%, PCo2 = 14.5%). Notably, the PE_N treatment exhibited a community composition relatively similar to the CK, while being distinct from the other treatments, suggesting that PE and N independently influence the consumer community composition. Similarly, in the phototroph community (Figure 3b), PCoA demonstrated a more pronounced separation among the treatments (R^2^ = 0.52, *p* < 0.001), with PCo1 and PCo2 accounting for 43.2% of the total variation (PCo1 = 25.0%, PCo2 = 18.2%). The CK and PE treatments clustered distinctly from the N and PE_N treatments, indicating that the N-containing conditions significantly influenced the phototroph community composition. For the parasite community (Figure 3c), the PCoA results also showed significant treatment-related differences (R^2^ = 0.60, *p* < 0.001), with PCo1 and PCo2 explaining 64.4% of the total variation (PCo1 = 43.8%, PCo2 = 21.6%). The treatments containing PE, especially PE_N, exhibited distinct clustering patterns compared to the CK treatment, suggesting that the PE_N treatment substantially altered the parasite community structure.

**Figure 4:**
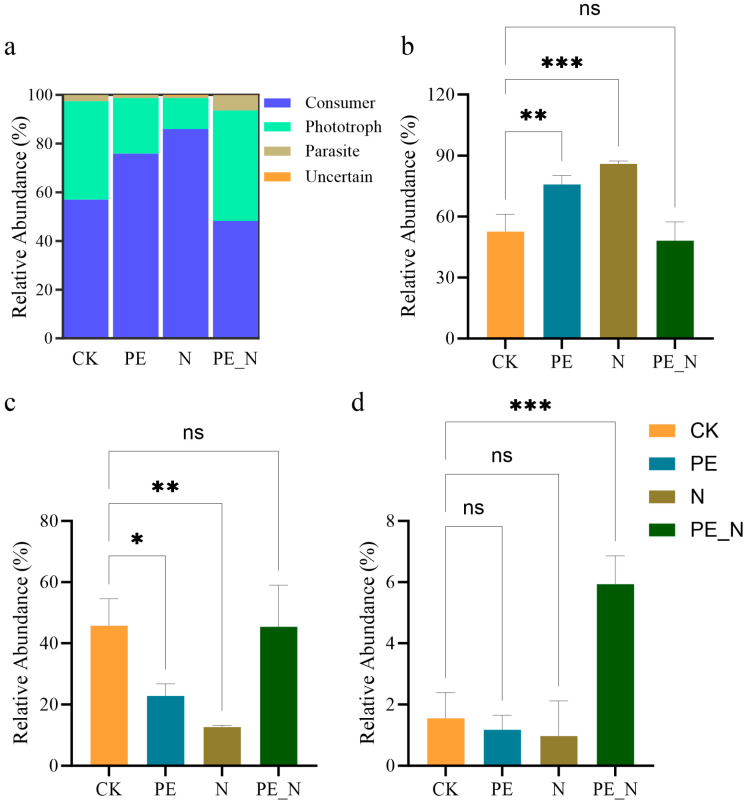
Relative abundance of dominant functional protist (**a**) and varied analysis of consumers (**b**), phototrophs (**c**), and parasites (**d**) under different treatments. The ns indicates no significant difference and asterisk (*) indicates a significant difference, according to the Kruskal-Wallis test with the Dunn post-hoc test, with the significance levels defined as follows: * *p* < 0.05, ** *p* < 0.01 and *** *p* < 0.001.

The relative abundance of dominant functional groups also varied significantly across the treatments (Figure 4a). Consumers were the most dominant functional group regardless of the treatment, followed by phototrophs, while parasites and unidentified groups were less abundant. The relative abundance of consumers differed significantly among the treatments (Figure 4b). Both the PE and N treatments significantly increased the consumer abundance compared to the CK (*p* < 0.01, *p* < 0.001, respectively). Although the PE_N treatment showed a slightly lower consumer abundance than the N treatment, the difference was not statistically significant. Phototroph abundance displayed distinct patterns under the different treatments (Figure 4c). Both the PE and N treatments significantly reduced the phototroph abundance relative to the CK (*p* < 0.05 and *p* < 0.01, respectively), whereas the PE_N treatment showed no significant differences compared to the CK. Parasite abundance was significantly affected by these treatments (Figure 4d). The PE_N treatment significantly increased the parasite abundance compared to all the other treatments (*p* < 0.001), whereas there were no significant differences in the parasite abundance among the CK, PE, and N treatments.

### 3.5. Contributions of Protist Community and Soil Characteristics in Rape Biomass

**Figure 5:**
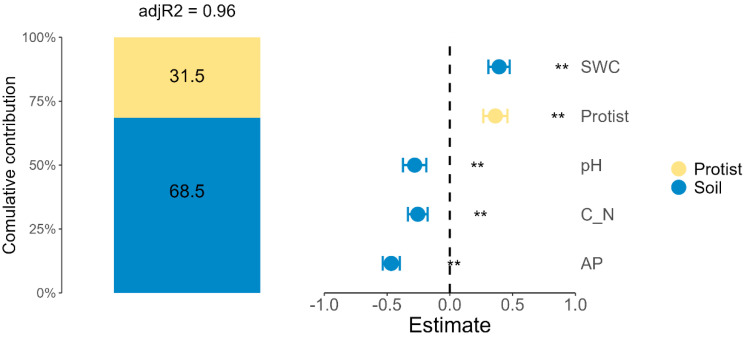
Parameter estimates and variance explained rape biomass. Parameters were classified into two groups: protist community composition (protist) and soil characteristics (soil). SWC stands for soil water content; C/N represents the ratio of total carbon to total nitrogen; AP stands for available phosphorus content. Significance levels defined as follows: ** *p* < 0.01.

The generalized linear mixed model analysis demonstrated that the soil properties and the protist community composition together explained 96% of the variation in the rape biomass (Figure 5, adj R^2^ = 0.96). Among these, the soil properties emerged as the primary contributors, accounting for 68.5% of the total variation, while the protist community composition explained the remaining 31.5%. Within the soil properties, the SWC showed a significant positive correlation with the rape biomass (*p* < 0.01), whereas the soil pH, the C/N ratio, and the AP exhibited significant negative correlations with biomass (*p* < 0.01). Similarly, protist community composition was positively correlated with the rape biomass (*p* < 0.01).

## 4. Discussion

### 4.1. Impacts of PE and Nitrogen Fertilization on Soil Nutrient Dynamics and Regulation Strategies

This study demonstrates the substantial impact of polyethylene (PE) microplastic contamination on soil nutrient dynamics, particularly when combined with nitrogen fertilization (PE_N). Notable disruptions included reductions in the SWC, alterations to the C/N balance and decreases in nutrient availability, such as the AP (Table 1). Specifically, the SWC was markedly reduced under the PE and PE_N treatments, while the AP levels were strongly suppressed under the PE_N treatment. Although nitrogen fertilization (N) improved water retention, it only partially mitigated the negative effects of PE contamination. The interplay between PE contamination and soil nutrient cycling underscores its dual impact on the physical and biochemical processes essential for soil fertility [46].

The observed reduction in the SWC under PE-containing treatments can be attributed to the hydrophobic properties of PE particles, which obstruct soil pores and decrease porosity, thereby compromising the water retention capacity [47]. These structural modifications restrict water movement and generate heterogeneous moisture distribution, ultimately impacting nutrient solubility and mobility, particularly for moisture-dependent nutrients such as phosphorus [48]. The PE_N treatment exacerbated these effects through altered soil aggregation and disrupted root-zone water connectivity [28]. Additionally, PE contamination significantly elevated the soil total carbon and the C/N ratios (Table 1), indicating altered organic matter decomposition patterns. The increased C/N ratio under the PE treatment suggests a shift in microbial metabolism toward labile carbon degradation, rather than nitrogen mineralization [49]. This metabolic shift disrupts nutrient cycling, reducing NH_4_^+^-N and NO_3_^−^-N availability under the PE treatment. Furthermore, the diminished soil pH buffering capacity under PE exposure likely compromised phosphorus solubilization and transport, intensifying AP deficiencies [50].

The strong association between PE contamination and disrupted nutrient dynamics aligns with prior research indicating that microplastics alter microbial community function, reducing microbes’ efficiency in mediating nutrient turnover [51]. Nitrogen fertilization partially mitigated these effects, as evidenced by the increased inorganic nitrogen levels under the PE_N treatment [52,53]. Nitrogen inputs likely stimulate microbial activity, supporting the transformation between organic and inorganic nitrogen forms [54]. However, these interventions were insufficient to fully counteract the severe nutrient imbalance caused by PE contamination. Beyond its impacts on soil properties, PE contamination directly interferes with plant root systems by adhering to root surfaces and hindering development [55]. This physical barrier disrupted the connectivity between the roots and soil, reducing nutrient uptake and water absorption. Moreover, degradation products from PE, such as phthalates and bisphenol A, introduce phytotoxic effects, inhibiting seed germination and suppressing root elongation [56]. These combined effects diminish the absorption of key nutrients, including nitrogen (N), phosphorus (P), and potassium (K), ultimately impairing chlorophyll synthesis, photosynthetic efficiency, and plant productivity [57]. To mitigate the adverse effects of PE contamination, integrating organic amendments, such as compost or biochar, could enhance the soil structure, improve water retention, offset PE’s hydrophobic effects, and stimulate microbial activity [58]. Additionally, combining slow-release phosphorus fertilizers with organic amendments may provide a synergistic solution by increasing the AP availability and improving the overall nutrient profile [59]. Long-term studies are essential to address the cumulative effects of persistent PE contamination on soil ecosystems and to better understand PE’s interactions with microbial processes and nutrient cycling.

### 4.2. Impacts of PE and Nitrogen Fertilization on Soil Protist Diversity and Functional Groups

This study investigated the effects of PE, N, and their combined treatment, PE_N, on soil protist diversity, community composition, and functional groups [60]. Key findings revealed that the protist diversity (measured by Shannon and Chao1 indices) increased under the individual PE and N treatments but remained unchanged under the PE_N treatment (Figure 2). The functional groups, including consumers, phototrophs, and parasites, exhibited distinct responses. Specifically, consumer abundance increased under the PE and N treatments, while phototrophs declined. Notably, the PE_N treatment restored these groups to levels comparable to the CK, indicating that nitrogen addition partially mitigated PE-induced shifts in the community structure (Figure 3 and Figure 4).

Protist diversity serves as a critical indicator of the soil ecosystem’s health and functional capacity [57]. The increased Shannon and Chao1 indices under the PE and N treatments likely reflect enriched soil conditions; PE introduces carbon substrates, supporting microbial proliferation [61], while nitrogen fertilization boosts microbial activity through nutrient enrichment [52]. The functional group dynamics further illuminated shifts in soil trophic interactions. The elevated consumer abundance under the PE and N treatments (Figure 3) may stem from enhanced prey availability (e.g., bacteria and fungi) fueled by PE-derived carbon or nitrogen-stimulated nutrient cycling [33,62,63]. Conversely, the reduced phototroph abundance (algae dominated) under the PE and N treatments may result from water deficits (PE-induced hydrophobicity) and altered nutrient stoichiometry [54,64].

The recovery of consumer and phototrophic abundances to the CK levels in the PE_N-treated soils suggests that nitrogen alleviates PE-driven stressors. Nitrogen-stimulated bacterial growth likely rebalances trophic interactions, reactivating consumer-mediated nutrient recycling through predation on bacteria and other protists [33,65]. Although this facilitated nutrient turnover, the phototrophs remained vulnerable to water stress under the PE_N treatment because the reduced SWC impaired photosynthetic activity [66]. Nitrogen fertilization served multiple functions in this context: it improved the soil quality [67], supported consumer protist populations [54], and enhanced nutrient availability and chlorophyll synthesis, contributing to stabilized phototrophic protist abundance in the PE_N-treated soils [66]. In contrast, PE alone primarily stimulates consumer protists through the introduction of recalcitrant carbon sources [31], leading to trophic imbalances and decreased phototroph abundance. These findings underscore the crucial role of consumer protists in maintaining soil nutrient and carbon cycling under stress conditions within microbial food webs.

### 4.3. Main Drivers of Rape Growth Parameters and Biomass

All the treatments significantly influenced the rapeseed growth and biomass (Figure 1 and Figure 5). The PE treatment markedly reduced the plant biomass, leaf chlorophyll content, and root activity compared to the CK, while nitrogen fertilization enhanced these parameters. Although the combined PE_N treatment partially mitigated PE’s negative effects, the plant growth indicators remained below those observed under the N-only and CK treatments (Figure 1). These findings suggest that PE contamination impairs plant growth through alterations in key soil physicochemical and biological properties, while nitrogen fertilization helps buffer these adverse impacts.

Nitrogen fertilization mitigated the effects of PE contamination through two primary mechanisms. First, it provides readily available nutrients (NH_4_^+^-N and NO_3_^−^-N), compensating for PE-induced nutrient deficiencies [68]. This direct nutrient supplementation supported chlorophyll synthesis and the photosynthetic capacity, which are processes critical for biomass accumulation [68,69,70]. The observed improvements in the chlorophyll content and the plant biomass under nitrogen fertilization reflect its essential role in maintaining plant metabolism under stress conditions [71]. Second, nitrogen addition indirectly stimulates soil biota activity, particularly microbial and protist communities, which are vital for nutrient cycling and soil structure enhancement [72]. Consumer protists, for instance, facilitate nutrient cycling through bacterial and fungal predation while improving root zone conditions, nutrient availability, and soil water retention [54]. As shown in Figure 5, this biological activation helped alleviate PE-induced disruptions in soil functions and supported rape growth. These results align with those of previous studies, showing that nitrogen inputs enhance the abundance and functionality of key soil microbial communities, thereby restoring ecosystem services in degraded soils [66,73].

However, the PE_N treatment failed to fully restore the rape growth parameters to the control levels, highlighting the complex stress imposed by PE contamination on both soil and plants. This dual stress encompasses physical (reduced soil water retention and impeded root penetration) and biological effects (disrupted microbial and protist communities) [29,74]. PE particles reduce the soil water content by increasing the void ratio and promoting evaporative water loss, creating water stress that limits nutrient solubility and plant availability [75]. Furthermore, PE contamination significantly affects protist functional groups, particularly phototrophs, which serve as primary producers in soil ecosystems. Under the PE_N treatment, phototrophic protist abundance declined significantly due to the reduced soil water content and increased competition with consumer protists for limited resources (Figure 5). Soil algae and other phototrophs contribute to carbon fixation, promote carbon cycling, and enhance soil oxygen availability [15,76]. Their reduced abundance restricts carbon inputs and negatively affects plant growth by limiting nutrient cycling and biogeochemical processes [66].

Figure 5 illustrates the critical interaction between soil properties and protist communities in determining the rape biomass, with soil water content emerging as a primary driver. Phototrophic protists show high sensitivity to changes in water availability, while consumer protists regulate microbial food web dynamics, affecting energy flows [15,46,72]. The decline in phototrophs under the PE and N treatments exacerbated carbon and nutrient limitations, reducing plant productivity. Conversely, the partial recovery of phototrophic protists under the PE_N treatment correlated with improved plant productivity. These results emphasize that nitrogen fertilization influences soil nutrient levels and functional protist communities, thereby helping to alleviate the effects of microplastics on plant growth.

In summary, this study provides compelling evidence of the intricate interactions between polyethylene microplastic (PE) contamination and nitrogen fertilization in agricultural systems, specifically focusing on their effects on soil protist communities and the productivity of rape (*Brassica napus* L.). This research highlights the need for long-term, in situ field studies to understand the microplastic effects in real agricultural environments and develop sustainable soil management practices that address the growing challenge of microplastic contamination in agricultural systems.

## 5. Conclusions

Through high-throughput sequencing and comprehensive soil–plant trait analyses based on a pot experiment, several significant findings emerged. PE contamination alone negatively impacted the soil properties and the crop growth by reducing the soil water retention capacity and the rape biomass, while increasing the TC, C/N ratios, and NH₄⁺-N/NO₃^−^-N levels. These alterations disrupted biogeochemical cycles, particularly carbon and nitrogen cycling, ultimately constraining plant growth and productivity. In contrast, nitrogen fertilization significantly improved the crop performance, enhancing the rape biomass and the chlorophyll content through increased nutrient availability. Remarkably, the combined PE_N treatment demonstrated antagonistic effects. The protist diversity and functional group composition stabilized to levels comparable to the control, and the rape biomass showed no significant difference from the control group, despite substantial reductions under the PE treatment alone. Soil nutrient dynamics (e.g., the SWC and the C/N ratio) and the protist community structure collectively explained 96% of the observed biomass variation. These findings highlight the potential of nitrogen fertilization as an effective mitigation strategy for microplastic-induced soil degradation. 

## Figures and Tables

**Table 1:** Soil physicochemical characteristics.

Treatment	SWC	pH	TC (g/kg)	TN (g/kg)	C/N	AP (mg/kg)	NH_4_^+^-N (mg/kg)	NO_3_^+^-N (mg/kg)
CK	0.23 ± 0.02 ^a^	6.64 ± 0.11 ^ab^	48.13 ± 2.7 ^c^	5.71 ± 0.19 ^a^	8.40 ± 0.83 ^c^	28.51 ± 3.47 ^a^	10.32 ± 0.45 ^b^	10.64 ± 0.52 ^c^
PE	0.20 ± 0.02 ^ab^	6.74 ± 0.20 ^a^	60.80 ± 3.4 ^ab^	5.65 ± 0.08 ^a^	10.53 ± 0.51 ^ab^	23.57 ± 0.12 ^b^	10.71 ± 0.44 ^ab^	10.56 ± 0.48 ^c^
N	0.24 ± 0.01 ^a^	6.30 ± 0.08 ^b^	52.41 ± 4.7 ^c^	5.77 ± 0.11 ^a^	9.14 ± 1.51 ^c^	24.65 ± 0.46 ^b^	10.82 ± 0.24 ^ab^	13.17 ± 0.64 ^b^
PE_N	0.16 ± 0.01 ^b^	6.45 ± 0.08 ^ab^	63.39 ± 4.4 ^a^	5.68 ± 0.05 ^a^	11.03 ± 2.09 ^a^	21.57 ± 0.18 ^c^	11.98 ± 0.42 ^a^	15.04 ± 0.40 ^a^

Note: Values represent means ± SE (n = 4). Different letters indicate significant differences. SWC stands for soil water content; TC stands for total carbon content; TN stands for total nitrogen content in soil; C/N represents the ratio of total carbon to total nitrogen; AP stands for available phosphorus content; NH_4_^+^-N represents ammonium nitrogen content; NO_3_^+^-N represents nitrate nitrogen content.

## Data Availability

The original contributions presented in this study are included in the article. Further inquiries can be directed to the corresponding author.
